# *Arabidopsis* HSFA9 Acts as a Regulator of Heat Response Gene Expression and the Acquisition of Thermotolerance and Seed Longevity

**DOI:** 10.1093/pcp/pcad164

**Published:** 2023-12-20

**Authors:** Xiaohua Wang, Yan Zhu, Ling Tang, Yuanyuan Wang, Runze Sun, Xin Deng

**Affiliations:** State Key Laboratory of Plant Diversity and Specialty Crops, Institute of Botany, Chinese Academy of Sciences, Beijing 100093, China; China National Botanical Garden, Beijing 100093, China; State Key Laboratory of Plant Diversity and Specialty Crops, Institute of Botany, Chinese Academy of Sciences, Beijing 100093, China; China National Botanical Garden, Beijing 100093, China; State Key Laboratory of Plant Diversity and Specialty Crops, Institute of Botany, Chinese Academy of Sciences, Beijing 100093, China; China National Botanical Garden, Beijing 100093, China; University of Chinese Academy of Sciences, Beijing 100049, China; State Key Laboratory of Plant Diversity and Specialty Crops, Institute of Botany, Chinese Academy of Sciences, Beijing 100093, China; China National Botanical Garden, Beijing 100093, China; University of Chinese Academy of Sciences, Beijing 100049, China; State Key Laboratory of Plant Diversity and Specialty Crops, Institute of Botany, Chinese Academy of Sciences, Beijing 100093, China; China National Botanical Garden, Beijing 100093, China; State Key Laboratory of Plant Diversity and Specialty Crops, Institute of Botany, Chinese Academy of Sciences, Beijing 100093, China; China National Botanical Garden, Beijing 100093, China

**Keywords:** Deterioration tolerance, Gene expression, Heat shock transcription factors, Seed longevity, Thermotolerance

## Abstract

Heat-shock transcription factors (HSFs) are crucial for regulating plant responses to heat and various stresses, as well as for maintaining normal cellular functions and plant development. HSFA9 and HSFA2 are two of the *Arabidopsis* class A HSFs and their expressions are dramatically induced in response to heat shock (HS) stress among all 21 *Arabidopsis* HSFs. However, the detailed biological roles of their cooperation have not been fully characterized. In this study, we employed an integrated approach that combined bioinformatics, molecular genetics and computational analysis to identify and validate the molecular mechanism that controls seed longevity and thermotolerance in *Arabidopsis*. The acquisition of tolerance to deterioration was accompanied by a significant transcriptional switch that involved the induction of primary metabolism, reactive oxygen species and unfolded protein response, as well as the regulation of genes involved in response to dehydration, heat and hypoxia. In addition, the *cis*-regulatory motif analysis in normal stored and controlled deterioration treatment (CDT) seeds confirmed the CDT-repressed genes with heat-shock element (HSE) in their promoters. Using a yeast two-hybrid and molecular dynamic interaction assay, it is shown that HSFA9 acted as a potential regulator that can interact with HSFA2. Moreover, the knock-out mutants of both HSFA9 and HSFA2 displayed a significant reduction in seed longevity. These novel findings link HSF transcription factors with seed deterioration tolerance and longevity.

## Introduction

Seeds, during their development, acquire a set of remarkable protective mechanisms that are essential to resist extreme desiccation and temperature conditions which are crucial for field yield, seed maturation and dormancy, germination and seedling establishment. These mechanisms include high- and low-temperature resistance, desiccation tolerance (DT) or the ability to withstand complete drying and rehydration and seed longevity (also referred as storability or vigor) (Zinsmeister et al. 2020). Seed longevity, defined as the ability of seeds to remain viable over a certain period of time, is an important trait in agronomy, food security and the conservation of genetic diversity. There is substantial evidence suggesting that the acquisition of seed longevity is a multifunctional trait, with a variety of mechanisms potentially contributing to this complex regulatory network (Verdier et al. 2013, Gonzalez-Morales et al. 2016). In angiosperm seeds, the processes of DT, heat shock resistance, seed maturation, dormancy and longevity all involve the expression of overlapping gene sets and the complex regulatory network that influence cell survival during dry conditions. These genes encode protective molecules, such as late embryogenesis-abundant (LEA) proteins (Manfre et al. 2008) and heat-shock proteins (HSPs), various enzymes involved in scavenging reactive oxygen species (Bailly 2004) and biosynthesis of a set of antioxidant compounds against oxidative stress such as glutathione, tocopherols and flavonoids (Sano et al. 2016, Leprince et al. 2017, Zinsmeister et al. 2020, Song et al. 2023).

In plants, the heat-shock response, seed maturation, DT and longevity processes are mainly controlled by a large set of transcription factors (TFs). Several transcriptional activators have been identified, such as the LAFL network, including LEAFY COTYLEDON 1 (LEC1), ABSCISIC ACID INSENSITIVE 3 (ABI3), FUSCA 3 (FUS3) and LEAFY COTYLEDON 2 (LEC2), that plays a role in controlling seed maturation (Santos‐Mendoza et al. 2008, Gechev et al. 2021) and more specific HSFs (Andrasi et al. 2021). Overexpression of HaHSFA9, a heat-shock factor from sunflowers, in transgenic tobacco has been shown to increase the accumulation of HSPs, and consequent improvement of seed thermotolerance and resistance to controlled deterioration treatment (CDT) (Prieto-Dapena et al. 2006). Additional phenotypes, such as exceptional protection of the photosynthetic apparatus from dehydration and oxidative stress in tobacco seedlings (Almoguera et al. 2015) and the ability to tolerate severe water loss (Prieto-Dapena et al. 2008), were also observed in the HaHSFA9 overexpression lines. In *Arabidopsis*, the expression of HSFA9 is shown to be regulated under a seed-specific TF, ABI3in seed-late maturation stage, which thus regulates seed dormancy and DT (Kotak et al. 2007). In *Medicago truncatula (M. truncatula)* seeds, the acquisition of DT and longevity was also linked to a regulatory network involving ABI3 and HSFA9 (Verdier et al. 2013). The DELAY OF GERMINATION (DOG) 1, originally identified for its involvement in seed dormancy, is also involved in seed longevity, as HSFA9 was down-regulated in the *dog1-1* mutant during seed-late maturation (Dekkers et al. 2016). One important factor that regulates seed dormancy and germination is the hormone abscisic acid (ABA). The *aba1-1* mutant, which has a deficiency in the amount of ABA, showed severe dormancy and longevity phenotypes, suggesting that ABA plays an important role in regulating both of these processes (Ooms et al. 1993). Recent evidence has shown that ABI5 plays a significant role in the longevity of seeds in *M. truncatula*, whereas *Arabidopsis* seed longevity was not affected in the *abi5* mutants (Zinsmeister et al. 2016). Several lines of evidence suggest that HSFA9 links the DT, seed dormancy and longevity signaling network with ABA signal pathway during seed development.

Understanding the genetic and molecular functions of HSFs that regulate seed germination vigor and longevity is particularly important to improve fundamental knowledge in this field and provide practical strategies for prolonging seed longevity. Although several studies have been conducted on seed longevity and regulatory mechanisms in sunflowers(Prieto-Dapena et al. 2006), tobacco (Almoguera et al. 2015), *Arabidopsis* (Kotak et al. 2007, Ninoles et al. 2022) and legumes (Zinsmeister et al. 2020), the regulatory mechanisms that activate seed vigor and longevity are still largely unknown.

In this study, we employed an integrated approach that combined transcriptomic, molecular genetics and molecular dynamics simulation analysis to identify and validate the regulatory mechanism of HSFA9 and HSFA2. To investigate the global transcriptional changes during controlled deterioration treatment, we performed RNA-sequencing (RNA-seq) and identified differentially expressed genes between the control and the CDT group. Through differential expression, gene enrichment and *cis*-elements enrichment analysis, we identified potential positive and negative regulators highly correlated with CDT suppression on seed longevity that may be manipulated by AtHSFA9. We further confirmed the HSFA9 and HSFA2 interaction with an *in vivo* binding experiment and *in silico* computational analysis. Hence, our findings provide new insights into the mechanisms of the seed vigor and longevity regulated by HSFA9 at the molecular level, and will serve as a valuable resource for molecular breeding aimed at improving heat and deterioration tolerance.

## Results

### HSFA9 is seed-specific and related to seed vigor and longevity

**Fig. 1:**
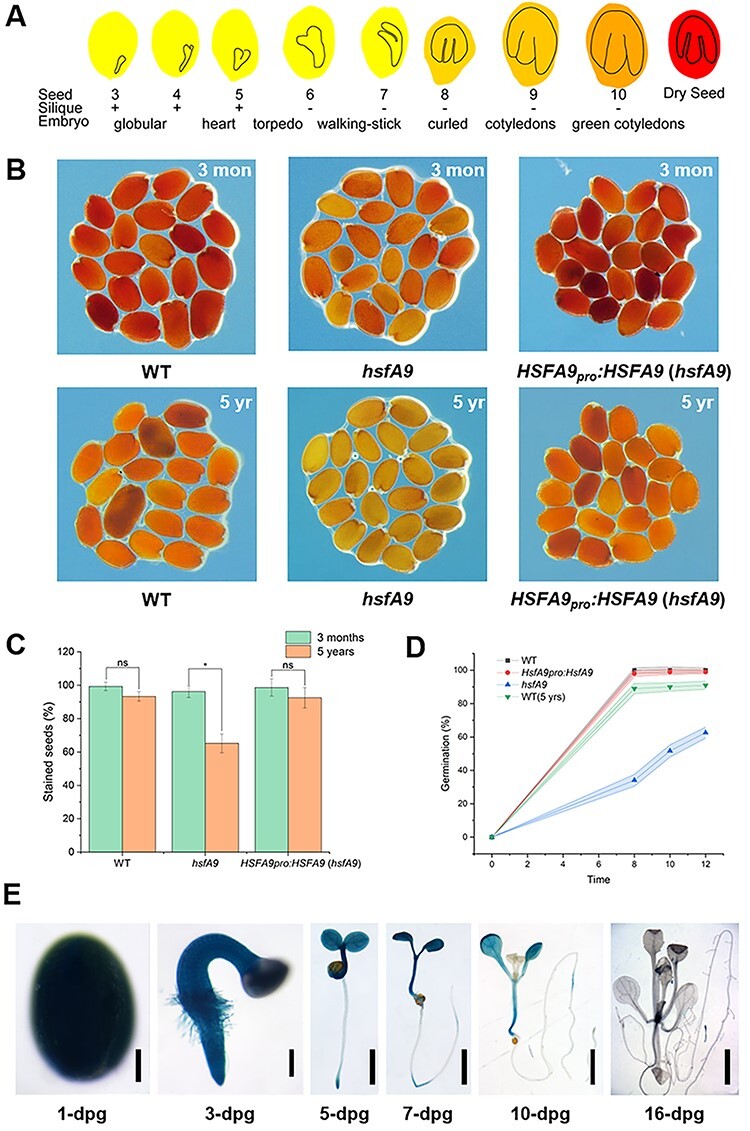
Characterization of seed vigor in the wild-type and *hsfA9* mutant. (A) Schematic representation of absolute expression of HSFA9 in *Arabidopsis* seed development. (B) Tetrazolium assay for seed viability of WT, *hsfA9* and *hsfA9* complemented lines stored for 3 months (upper panel) and 5 years (lower panel). (C) Bar plot showing the percentage of stained (red) seed. (ns means no significant, * means *P* < 0.05, *t*-test compared between the seeds stored for 3 months and 5 years). (D) Percentage germination rate observed at different times. Each time point represents average values obtained at different hours after post-inoculation. (E) GUS-staining assay of HSFA9 expression in HSFA9pro::GUS plants. Bars = 100 μm (1-dpg), 20 μm (3-dpg) and 0.2 mm (7- to 16-dpg).

A number of reports have indicated that the HSFA9 TFs display seed-specific expression (Kotak et al. 2007, Tejedor-Cano et al. 2010). It accumulates during seed maturation and reaches the highest level in dry seeds (**Fig. 1A**). To investigate the effect of HSFA9 on seed vigor and longevity, a triphenyltetrazolium chloride (TTC) staining was carried out with the *hsfA9* mutant and a native promoter-driven HSFA9 complementary line that were harvested 3 months and 5 years ago, respectively, and stored at room temperature with silica gel dryer. TTC precipitates to red colored 2,3,5-triphenyl formazan by the activity of dehydrogenases present only in the living cells, whereas it remains unstained in dead seeds (**Fig. 1B**). Almost all of the seeds harvested after 3 months had a dark red stain indicating their viability in wild-type, *hsfA9* and *hsfA9* complementary lines (**Fig. 1B**). The *hsfA9* seeds lost their vigor dramatically with a 65.2% TTC staining rate after 5 years of storage, while the wild-type and *hsfA9* complementary seeds still remained with a 93% TTC staining rate (**Fig. 1C**). In agreement, wild-type and *hsfA9* complementary seeds exhibited 100% germination; however, the *hsfA9* seeds showed <60% germination after 5 years of storage (**Fig. 1D**).

We further examined the tissue expression patterns for HSFA9 using transgenic *Arabidopsis* plants that expressed β-glucuronidase (GUS) under the control of the native promoter. As shown in **Fig. 1E**, the HSFA9 level declines rapidly during seed germination. Strong GUS staining was detected in the seed, radicle and cotyledons, while no GUS staining was observed in true leaves and roots after 16 days post-germination (**Fig. 1E**).

### The *hsfA9* seeds show reduced resistance to controlled deterioration treatment

**Fig. 2:**
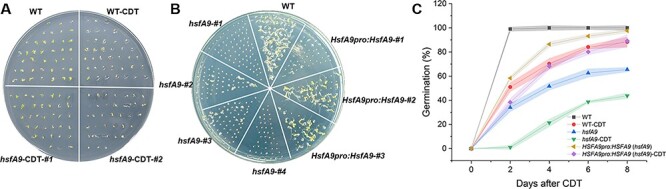
The role of HSFA9 in the acquisition of seed longevity during CDT. (A) Analysis of germination efficiency of wild-type and *hsfA9* seed under normal and CDT conditions. #1 and #2 represent two different lines for *hsfA9* mutant. (B) The *hsfA9* complemented seeds showing the recovery of CDT tolerance. #1, #2, #3 and #4 represent different lines. (C) Germination plot of the seeds from WT, *hsfA9* and HSFA9 overexpression. Each time point represents average values obtained days after CDT treatment.

To further evaluate the role of AtHSFA9 in the process of seed longevity, seeds were subjected to CDT and the germination rate was analyzed. Wild-type seeds showed ∼80% germination whereas only <60% and <40% germination was observed in *hsfA9* and *hsfA9* lines after CDT treatment, respectively (**Fig. 2A**,**C**). On the contrary, wild-type and *hsfA9* seeds exhibited ∼100% germination without CDT treatment. Seed germination of *hsfa9* was rescued to 98% in the *hsfA9*-complemented lines under normal conditions (**Fig. 2B,C**). Interestingly, after 6 days of CDT, the *hsfA9*-complemented seeds exhibited a slight enhanced germination rate (85%) compared to WT seeds (80%) (**Fig. 2C**).

### Transcriptional profiles of wild-type and controlled deterioration seeds during germination

To identify the genes that are involved in HSFA9-mediated seed germination and longevity, we collected the seeds and carried out high-throughput sequencing of wild-type and *hsfA9* mutant under normal CDT after 6 h germination. Using Illumina sequencing, we obtained a total of 422.4 million of clean reads, which amounted to 63.4 Gb of base pairs. Over 96% and 92% of the clean reads had Phred-scaled quality scores at the Q20 and Q30 levels, respectively (error < 0.02%). The expression level of each gene was calculated by mapping the sequences to the reference genome and based on the fragments per kilobase of the transcript per million fragments mapped (FPKM) value of each transcript. Based on the gene expression data, all differential expressed genes (DEGs) were visualized via a heatmap for hierarchical cluster analysis of transcription abundance in normal and CDT conditions (**Supplementary Fig. S1A**). The heatmap of the expression profile matrix of DEGs between WT and *hsfA9* showed similar transcriptome profiles. It is worth noting that some transcripts between WT-CDT and *hsfA9*-CDT showed slightly different expression patterns involved in the CDTs, and a large number of DEGs showed higher expression levels in the *hsfa9* mutant under CDT condition, indicating a repression effect of HSFA9 on CDT-induced gene expression (**Supplementary Fig. S1A**). Additionally, we used RT-qPCR to confirm the CDT-induced HSP17.6 genes, which encodes a HSP related to heat-shock and seed development (**Supplementary Fig. S1B**), as well as CDT-suppressed *PAR1* gene, encoding a PHYTOCHROME RAPIDLY REGULATED1 related to seedling development (**Supplementary Fig. S1C**). The fold-change observed with RT-qPCR was comparable to that seen in the RNA-seq data.

**Fig. 3:**
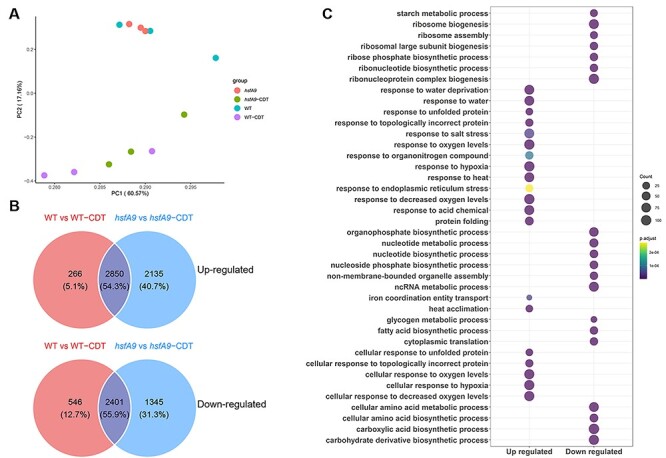
Expression profiles of DEGs in seeds during CDT. (A) PCA of gene expression profiles indicated that all the samples were divided into two distinct groups. (B) Venn diagram illustrating the up- and down-regulated genes between comparisons of WT vs WT-CDT and *hsfA9* vs *hsfA9*-CDT. (C) GO enrichment analysis of the up- and down-regulated genes across the comparison between WT and hsfA9 mutants under CDT. Dot color represents log10 (-adjust *P*-value) and dot size represents the number of genes.

Furthermore, the results of principal component analysis (PCA) showed that all the samples were separated into two distinct groups (as shown in **Fig. 3A**), which is consistent with the results obtained from the DEGs profile mentioned earlier. Using *q*-values of less than 0.01 for significantly different expression and at least 2-fold change, we identified a total of 3,116, 4,709, 4,985 and 1,942 genes that were up-regulated in WT versus WT-CDT, WT versus *hsfA9, hsfA9* versus *hsfA9*-CDT and WT-CDT versus *hsfA9*-CDT, respectively (**Fig. 3B**). Notably, a lower number of genes were down-regulated in WT versus WT-CDT (2947), WT versus *hsfA9* (3418) and *hsfA9* versus *hsfA9*-CDT (3746), respectively (**Fig. 3B**).

**Fig. 4:**
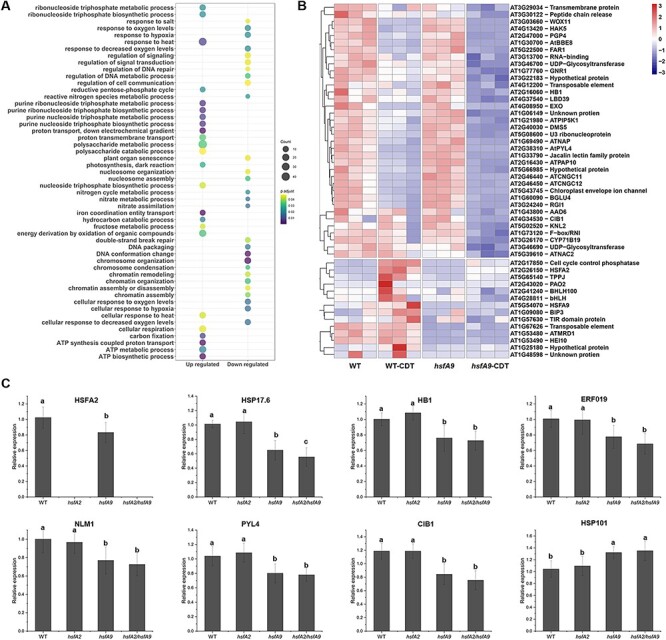
Analysis of differential expressed genes between WT and *hsfA9* under CDT. (A) Go enrichment analysis of the significantly up- and down-regulated genes between comparisons of WT-CDT and *hsfA9*-CDT. Dot color represents log10 (-adjust *P*-value) and dot size represents the number of genes. (B) Heatmap of expression patterns for the top 50 CDT-suppressed genes. Hierarchical clustering analysis was performed for both samples and genes using the Euclidean distance as the similarity metric. (C) RT-qPCR analysis of eight representative genes (*HSFA2, HSP17.6, HB1, ERF019, NLM1, PYL4, CIB1* and *HSP101*) in response to CDT treatment in *hsfA2, hsfA9* and *hsfA2/hsfA9* mutants. Error bars show SD from three replicates. Letters in the bar plots indicating significant differences (P < 0.05).

There was more than a 50% overlap of differentially regulated gene sets between the WT versus WT-CDT and *hsfA9* versus *hsfA9*-CDT during CDT treatment (**Fig. 3B**). In total, 2,850 and 2,401 DEGs were identified as upregulated and down-regulated genes in these datasets, respectively. Following that, functional enrichment analyses of up- or down-regulated DEGs were conducted. The co-up-regulated DEGs were significantly enriched in terms of response to oxygen, heat, water and protein folding, as well as ER stress response, based on Gene Ontology (GO)-biological process (BP) analysis (**Fig. 4C**). In contrast, the down-regulated gene expression profile was enriched in ribosome biogenesis, RNA modification, lipid and starch metabolism (**Fig. 3C**). The GO enrichment showed functional overlap between these two datasets. The results showed that DEGs from the CDT treatment included significantly substantial genes associated with response to dehydration, heat and hypoxia in both the wild-type and *hsfA9* mutant.

### Comparison of CDT-induced and suppressed DEGs between WT and *hsfA9* lines

The gene sets induced by CDT in WT but not in *hsfA9* mutant are especially notable, as they might be responsible for seed vigor and longevity changes associated with the malfunction of *HSFA9*. A total of 1,942 genes showed significant differential expression between wild-type and *hsfA9* seedlings under CDT treatment, including 1,731 up-regulated and 211 down-regulated genes. The most significantly up-regulated DEGs in the *hsfA9* mutant were putatively related to different GO terms such as response to heat, purine metabolism and cellular respiration (**Fig. 4A**). In contrast, the down-regulated DEGs were significantly enriched for the regulation of response to hypoxia, oxidative stress, chromosome organization and DNA repair (**Fig. 4A**). The top 50 down-regulated DEGs in *hsfA9*-CDT versus WT-CDT seedlings were demonstrated in subsets by heat maps (**Fig. 4B**). Interestingly, a gene encoding a heat-shock TFs A2 (HSFA2) was down-regulated (Log2FC 1.7) in *hsfA9*-CDT seedlings, reminding us that HSFA2 may also participate in seed-deterioration response. To examine how the suppressed DEGs during CDT treatment may exhibit different expression patters in *hsfA2, hsfA9* and *hsfA2 /hsfA9* mutants, eight genes representative genes were selected and assayed using RT-qPCR (**Fig. 4C**). The eight selected genes (*HSFA2, HSP17.6, HB1, NLM1, ERF019, PYL4, CIB1* and *HSP101*) represent a spectrum of functions, including responses to water stress, ABA signaling, aging-induced cell death and cellular reactions to hypoxia, light sensitivity and unfolded protein response, providing a comprehensive overview of HSFA9’s regulatory impact across diverse biological processes. In the mutant form of HSFA9, where *HSFA2, HSP17.6, HB1, NLM1, ERF019, PYL4* and *CIB1* showed reduced expression levels, it was especially notable that HSP101 displayed a distinct expression pattern, implying a unique regulatory response. Importantly, in the double mutant scenario involving both HSFA9 and HSFA2 knock-out, no significant effects on the expression levels of these genes were observed compared to the *hsfA9* mutant, except for HSP17.6. In accordance with RNA-seq data, our qPCR analysis demonstrated a 23% reduction in the expression of *HSFA2* in *hsfA9*-CDT compared to the wild-type seedlings (**Fig. 4C**). This consistency further supports the notion that HSFA2 likely plays a role in the seed deterioration response alongside HSFA9.

### *K*-means clustering of DEGs

**Fig. 5:**
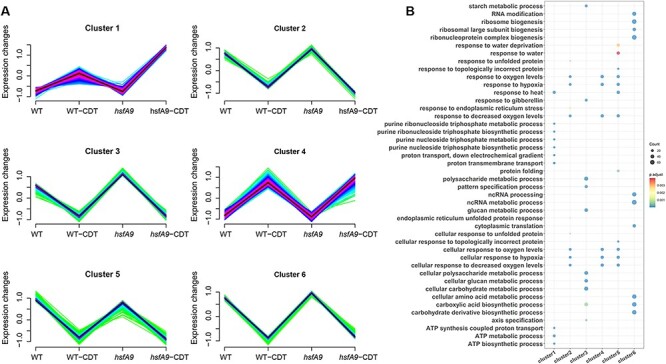
Gene expression patterns in response to controlled deterioration. (A) Expression patterns of genes in six clusters generated from a total of 5,521 DEGs. Gene numbers used for the analysis are shown in parentheses. (B) GO-enrichment results of biological process category for the genes in different clusters. Dot size represents the number of genes, and gradient color of the dot indicates the significance log10 (-adjust *P*-value) value of the gene set.

The *k*-means clustering analysis was next conducted on the 5,251 DEGs using the k-means clustering approach. **Fig. 5** illustrates that a total of six clusters with distinct gene expression dynamics were identified. Notably, four clusters displayed distinct expression patterns (**Fig. 5A**). A total of 1,054 genes in cluster 3 and 813 genes in cluster 5 showed higher expression levels in untreated condition than that during CDT condition. The other four clusters showed largely no difference between *hsfA9* and WT under untreated condition, but up-regulated (cluster1 and cluster 4) or down-regulated (cluster 2 and cluster 6) expressed pattern under CDT-treated condition. Among them, 784 and 744 genes in cluster 1 and cluster 4 showed higher expression (cluster 1) or similar (cluster 4) levels in *hsfA9*-CDT than that in WT-CDT, while 440 genes in cluster 2 and 1,207 genes in cluster 6 showed lower (cluster 2) or higher (cluster 6) levels in *hsfA9*-CDT than that in WT-CDT (**Fig. 5A**).

In order to better understand the functions of these genes, we conducted GO-enrichment analysis on six clusters. Cluster 1 was significantly enriched in ‘purine nucleoside triphosphate biosynthetic and metabolic process’, ‘ATP metabolic’, ‘proton transmembrane transport’ and ‘heat response’. Cluster 2 was highly associated with ‘oxygen levels/hypoxia’, ‘unfolded protein response’ and ‘endoplasmic reticulum stress’, whereas cluster_4 was exclusively overrepresented in items of ‘oxygen levels’. Cluster 3 was composed of genes enriched in ‘cellular glucan and polysaccharide metabolic’, ‘response to gibberellin’ and ‘pattern specification process’ that were down-regulated during CDT treatment in both wild-type and *hsfA9* mutant. Interestingly, cluster 5 was found to be significantly enriched in functional categories related to response to oxygen/hypoxia, water deprivation, heat and topologically incorrect protein. Additionally, genes in this cluster were not only significantly down-regulated by CDT treatment but also up-regulated by the *hsfA9* mutation in untreated conditions. In cluster 6, the gene expression pattern was mainly enriched in items such as ‘ribosome biogenesis’, ‘ncRNA processing’ and ‘cellular amino acid metabolic’ (**Fig. 5B**). These results could provide important cues for deciphering the molecular regulation of seed deterioration.

### Identification of WGCNA modules associated with CDT treatment

**Fig. 6:**
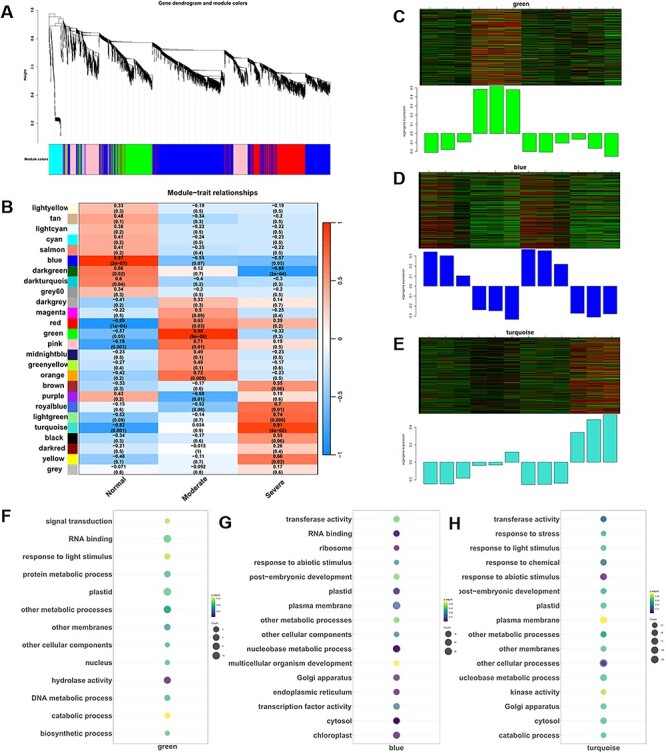
Weighted gene co-expression network analysis (WGCNA) of genes related to transcriptomic response during CDT treatment. (A) DEGs clustering and module screening based on gene expression pattern. The top panel displays the gene dendrogram, while the bottom panel exhibits the identified gene modules, each distinguished by a unique color. In total, 26 modules were discerned. (B) Module–trait correlation analysis. The left panel depicts the 26 identified modules, and the color scale on the right represents module–trait correlations ranging from −1 (blue) to 1 (red). The terms ‘normal’, ‘moderate’ and ‘aeveral’ indicate the relative seed germination rates following CDT treatment. (C)–(E) Heat maps illustrating the dynamic expression patterns of co-expressed genes in the ‘green’, ‘blue’ and ‘turquoise’ modules. (F)–(H) The GO-enrichment results of biological process category for genes within the three most significant modules. Dot size corresponds to the number of genes, and the gradient color of the dot indicates the significance as log10 (-adjust *P*-value) values.

To explore genes associated with the transcriptomic response during CDT treatment, we utilized a weighted gene co-expression network analysis (WGCNA) to identify gene modules between WT and *hsfA9* under normal and CDT conditions. WGCNA, performed with the previously identified 5,251 non-redundant DEGs, resulted in the identification of 26 WGCNA modules (**Fig. 6A**). Analysis of module-trait relationships revealed high correlations for module blue (*r* = 0.97, *P* = 2.0 × 10^–7^), green (*r* = 0.98, *P* = 9.0 × 10^–9^) and turquoise (*r* = 0.91, P = 4.0 × 10^–5^) with the germination trait under CDT treatment (**Fig. 6B**).

Subsequently, we conducted GO-term enrichment analysis for genes within these modules. In terms of biological processes, the genes in the green module showed significant enrichment in signal transduction, protein metabolic processes, metabolic processes, biosynthetic processes and catabolic processes (**Fig. 6C,F**). Enrichment results in **Fig. 6D,G** highlighted that the genes in the blue module were significantly enriched in organelle biogenesis (e.g. plasma membrane, Golgi apparatus, endoplasmic reticulum, chloroplast, ribosome and plastid), response to abiotic stimulus, post-embryonic development and metabolic processes. The module-trait heatmap in **Fig. 6B** indicated that the turquoise module played a crucial role in the germination rate of seeds under CDT. Moreover, significant enrichment in biological processes was observed in **Fig. 6E,H**, including responses to stress, response to abiotic stimulus, organelle development (such as plastid, Golgi apparatus and cytosol), membrane dynamics and metabolic processes.

### Identification of *cis*-elements enriched in the promoter regions of HSFA9-induced and -repressed genes

To better understand the possible regulatory *cis*-elements in the downstream genes affected by HSFA9, we scanned the *cis*-elements of the differentially regulated genes in the 2-kbp promoter regions. As shown in **Supplementary Fig. S2A**, in CDT induced (2,850) gene promoters, six most highly represented motifs were found to be ‘GAAGAAGAA’, ‘SAAAAAAAA (S=C/G)’, ‘CTCTCTCTCT’, ‘GMCACGTGKC (M=C/A, K=G/T)’, ‘GCCACGTCAGC’ and ‘TCGCCGGARA (R=G/A)’ (**Table 1**). Three motifs (ACGTGG, GAA-, AAA-rich) were enriched in CDT-induced genes with known function of ABA response, NRP1 response and REM19 binding in *Arabidopsis*. The best matched motif for CT-rich domain in *Arabidopsis* is ND_tnt.FRS9, a binding site of ND (no DNA-binding) TFs. The *cis*-elements enriched in CDT-repressed genes (2,401) were ‘AAAAAAAA’, ‘GAAGAAGAA’, ‘CACGTGGCA’, ‘AAACCCTAAA’, ‘AATAATAATAAWWW (W=A/T)’ and ‘TATATATATAYATAW (Y=T/C, W=A/T)’ (**Table 1**). Like that in CDT-induced promoters, motifs (ACGTGG, GAA-, AAA-rich) matched with ABA response, NRP1 response and REM19 binding, respectively, were closely related to ABI3-VP1 paralogs. Remarkably, AAT-box and AT-rich motif, specific binding sites of HSPs and heat-shock TFs were found in the promoters of CDT-repressed genes (**Supplementary Fig. S2B**).

**Table 1:** Overrepresented *cis*-elements in the promotors of CDT-induced and -repressed genes

	Rank	Discovered motif	E value	Name of matched motif in Arabidopsis	Target sequences with motif (%)
CDT induced	1	GAAGAAGAA	2.4e-25	BPC5	17.03
	2	SAAAAAAAA (S = C/G)	1.2e-18	REM19	18.76
	3	CTCTCTCTCT	1.9e-11	BPC1	14.03
	4	GMCACGTGKC (M = C/A, K = G/T)	2.4e-11	ABF2	7.65
	5	GCCACGTCAGC	1.0e-10	bHLH104	15.42
	6	TCGCCGGARA (R = G/A)	9.5e-009	Trihelix	4.73
CDT suppressed	1	AAAAAAAA	2.1e-028	REM19	17.05
	2	GAAGAAGAA	2.4e-18	BPC5	14.39
	3	CACGTGGCA	7.8e-15	ABI5	32.75
	4	AAACCCTAAA	6.6e-13	MYB related	19.97
	5	AATAATAATAAWWW (W = A/T)	7.8e-12	ATHB13	29.68
	6	TATATATATAYATAW (Y = T/C, W = A/T)	1.0e-11	ARID	13.73

The *cis*-elements in promoter of different cluster genes could be involved in the transcriptional regulation with different signaling pathways to control seed vigor and longevity. To figure out which kind of regulatory motifs were enriched in the promotors of DEGs in the six grouped clusters, we further performed the *cis*-element scanning and counting of the differentially regulated gene sets in the promoter regions. The matched *cis*-elements are found to be linked to a number of important TFs such as DREBs, BPCs, AP2/ERF, ARF, ABI3/ VP1, ESE, MYB, ABR, VRN, AS2, HSF, GATA and bZIP (**Table 2**).

**Table 2:** Overrepresented *cis*-elements in the promotors of DEGs in the grouped clusters

Grouped clusters	Discovered motifs	Name of matched motif in Arabidopsis	Occurrence in cluster genes with motif	*E*-value
Cluster 1	GAGAGAGA	BPC5	46.5	1.67e-39
	TTTTTTTT	ABI3VP1	47.8	2.64e-28
	CCGCCGCCG	ERF2	50.3	7.58e-16
	TGCCACGTG	ABF2	35.9	1.9e-11
	GAAGTTCTA	HSFA1E	15.42	2.31e-10
Cluster 2	GAGAGAGA	BPC5	65.05	7.65e-52
	CCTCCTCCT	TF3A	47.2	2.83e-48
	AAAAAAAAA	REM19	38.9	2.63e-30
	GGCGGCGGC	ERF9	34.3	1.31e-28
	CGGCGGCGG	RAP26	31.5	1.92e-16
Cluster 3	CTCTCTCTCT	BPC6	50.1	3.31e-69
	ATGCTGATG	GATA1	51.3	7.01e-51
	GGCGGCGGC	CRF4	50.4	2.87e-29
	TCATCATCA	ZML1	48.6	5.72e-23
	AGCGGCGGC	ESE3	41.2	2.28e-19
Cluster 4	GAGAGAGA	BPC1	65.7	3.53e-78
	CCGCCGCCG	SHN3	47.4	3.78e-53
	AAACCCTAA	TBP3	33.7	3.38e-49
	TGGCGGCGG	ABR1	52.9	1.29e-42
	ACGTCATCA	bZIP50	45.8	2.74e-25
Cluster 5	GAGAGAGA	BPC1	62.1	2.03e-65
	CTCTCTCT	FRS9	61.2	1.8e-47
	CCGCCGCCG	SHN3	53.6	3.84e-35
	TGGCGGCGG	ERF3	31.2	3.32e-34
	CATCATCAT	GATA15	30.1	2.35e-25
Cluster 6	GAGAGAGA	BPC5	52.1	7.48e-73
	TTTTTTTT	VRN1	46.9	4.03e-71
	CTCTCTCT	FRS9	46.8	1.15e-61
	CCGCCGCCG	SHN3	40.1	5.72e-39
	CCGTTTCAC	AS2	41.8	7.68e-35

The promoter analysis for gene sets in cluster 1 that were induced by CDT identified the most enriched *cis*-elements linked to TFs such as BPC5, ABI3VP1, ERF2, ABF2 and HSFA1E. The role of these linked TFs responses to diverse abiotic, heat and hypoxia/anoxia stress. In contrast, the motif analysis of gene sets in cluster_2 and cluster_3 which were suppressed by CDT-identified high enrichment of *cis*-elements including BPC5, TF3A, REM19, ERF9 and RAP26, BPC6, GATA1, CRF4, ZML1 and ESE3, respectively. These associated TFs mainly respond to seed development and dormancy. Moreover, the promoters in cluster_4 gene sets that were not responsible for CDT pose *cis*-elements associated with TFs including BPC1, SHN3, TBP3, ABR1 and bZIP50. These associated TFs are involved in the regulation of many aspects of plant growth and development. Similar putative *cis*-elements were also identified in the promoters of cluster 5 and 6 genes which are largely not regulated by HSFA9 and those that could be associated with TFs such as BPC, FRS9, SHN3, ERF3, GATA15 and AS2. Interestingly, these *cis*-element-linked TFs are mostly related to the regulation of different metabolism and hormone signaling such as auxin, GA and ethylene (**Table 2**).

### Effect of HSFA9 and HSFA2 on the persistence of seed vigor and thermotolerance

**Fig. 7:**
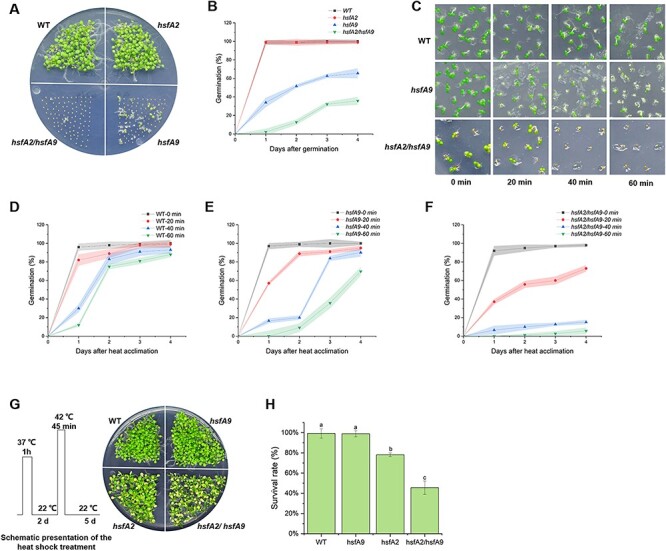
Seed longevity and basal thermotolerance are lost faster in *hsfA9/hsfA2* double mutants. (A) The *hsfA9/hsfA2* showing decreased germination efficiency under CDT, while *hsfA2* mutant still show CDT resistance. (B) Germination plot of the seeds from WT, *hsfA9, hsfA2 and hsfA9/hsfA2* lines. Each time point represents average values obtained from days after germination. (C) Malfunction of HSFA9 and HSFA2 leading to decreased germination efficiency compared to WT after heat-shock treatment at 55°C for 20, 40 and 60 min. Seeds of different lines were germinated on medium containing 0.5% MS medium for 5 days. (D)–(F) Germination plot of the seeds from WT, hsfA9 and hsfA2/hsfA9 under 20, 40 and 60 min heat-shock treatment. Each time point represents average values obtained from days after heat-shock. (F) The *hsfA9/hsfA2* seedlings do not tolerant to exposure to 37°C for 1 h, recover at 22°C for 2 days and heat-shock at 42°C for 45 min (see Scheme). (H) Survival rate of WT, *hsfA9, hsfA2 and hsfA9/hsfA2* plants after exposure to heat-shock. Error bars show SD from three replicates. Letters in the bar plots indicating significant differences (*P* < 0.05).

Wild-type and *hsfA2* seeds showed similar germination rates, indicating that HSFA2 is not directly involved in seed vigor under CDT (**Fig. 7A**). However, the germination rate of *hsfA2/hsfA9* double mutant seeds were approximately 30%, significantly lower than that of *hsfA9*, which had an average germination rate of 55–60% after 4 days (**Fig. 7B**). Since HSFA9 can induce the expression of HSPs as previously reported (Almoguera et al. 2009) and demonstrated through our transcriptome analysis, we examined whether the *HSFA9* mutation caused a defect in heat tolerance in comparison with the wild-type. The role of HSFA9 in basal thermotolerance was conducted by examining the seed germination that was exposed to 55°C for 20, 40 and 60 min (**Fig. 7C**). In a short heat-shock time of 20 min, 100% of the wild-type, 95% of *hsfA9* and 73% of *hsfA2* seeds germinated (**Fig. 7D****–****F**). It is noteworthy that wild-type and *hsfA9* seeds still retain their germination rate as high as 90% after heat-shock for 40 min. However, the germination rate of wild-type and *hsfA9* seeds was reduced to 85% and 63%, respectively, after heat-shock for 60 min (**Fig. 7D,F**). In contrast, the germination rate of the *hsfA2* seeds reduce to 5% after undergoing heat-shock for 60 min, suggesting a significant impact of the heat-shock treatment on the germination process specifically in the *hsfA2* mutant (**Fig. 7F**).

To further evaluate the role of HSFA2 and HSFA9 in acquired thermotolerance in seedlings, we subjected seedlings to a 1-h heat treatment at 37°C followed by a 2-day recovery period at 22°C (**Fig. 7G**). After the specified period of recovery, seedlings were then subjected to a higher heat-shock of 42°C for 45 min. The results show that the number of survival individuals of *hsfA2* mutants was fewer than for WT and *hsfA9* seedlings, and these seedlings displayed chlorisis (**Fig. 7G**). In contrast, *hsfA9* seedlings exhibited similar thermotolerant phenotypes as the WT. Loss-of-function mutants *hsfA2* displayed substantially decreased tolerance of heat-shock treatment compared with WT and *hsfA9* plants (**Fig. 7H**). However, the defects in both recovery and survival rate were dramatically lower in the *hsfA2/hsfA9* double mutant than in those of WT and *hsfA9* plants (**Fig. 7H**). These data suggest that HSFA9 may exert a partial effect on *hsfA2* in terms of seed vigor and seedling thermotolerance.

### Evidence for an interaction between HSFA9 and HSFA2

**Fig. 8:**
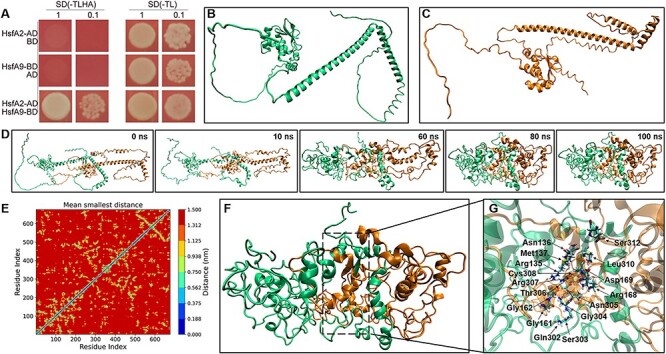
Heterodimerization of HSFA9 and HSFA2. (A) Y2H interaction assay between HSFA9 and HSFA2. Transformants were assayed for the activity of protein–protein interaction reporting genes by growth selective plating on -Trp -Leu -His -Ade SD medium. (B)-(C) The 3D model of HSFA9 and HSFA2 built by using Alphafold2. (D) Snapshots showing the oligomer formation between HSFA9 and HSFA2, where HSFA9 and HSFA2 are represented by green and orange colors, respectively. (E) The residue distant map between the Cα-atoms of each amino acid residue of HSFA9 and HSFA2. The scale adjacent to the distance map illustrates the distances between the residues. (F) The snapshot of the refined and stabilized HSFA9 and HSFA2 oligomer after 100 ns MD. (G) The cartoon view of the interface region and residues of HSFA9 and HSFA2 oligomer.

To determine whether an interaction can be formed between HSFA9 and HSFA2, we used the yeast two-hybrid system and molecular dynamics simulation. Initially, the *HSFA9* and *HSFA2* cDNA were incorporated in-frame into the GAL4 DNA-binding domain vector pGBKT7 (BD) and the GAL4 activation domain vector pGADT7 (AD), respectively. All of the constructed vectors were initially verified to confirm that they did not transactivate the *HIS3* and aminoimidazole ribonucleotide carboxylase 2 (*ADE2)* reporter genes. Yeast cells co-transformed with plasmids pGADT7-HSFA2 and pGBKT7, pGBKT7-HSFA9 and pGADT7, served as controls. Our results demonstrated that only yeast cells co-transformed with pGBKT7-HSFA2 and pGADT7-HSFA9 were able to grow on SD/-His/-Leu/-Trp/-Ade selective media (**Fig. 8A**), indicating a positive interaction between HSFA9 and HSFA2.

The structural properties and physical interaction between HSFA9 and HSFA2 were further confirmed by *in silico* analysis. The protein structures of HSFA9 and HSFA2 were initially predicted through Alphafold2. As shown in **Fig. 8B**, the predicted models of both HSFA9 and HSFA2 are dominated by random coil or intrinsically disordered secondary structure. On the other hand, the highly conserved DBD is represented with a small number of α-helices and β-sheets (**Fig. 8B**). To predict the physical interaction between HSFA9 and HSFA2, we performed a protein–protein docking study applying a rigid docking algorithm, in ZDOCK (http://zdock.umassmed.edu/). The best interaction complexes were ranked and selected based on the lowest free energy and highest cluster sizes (**Fig. 8C**). In order to evaluate the structural dynamic and stability of the HSFA9 and HSFA2 interaction complexes, we conducted a 100 ns all-atom molecular dynamics (MD) simulation for the top-docked structures generated with ZDOCK.

**Fig. 9:**
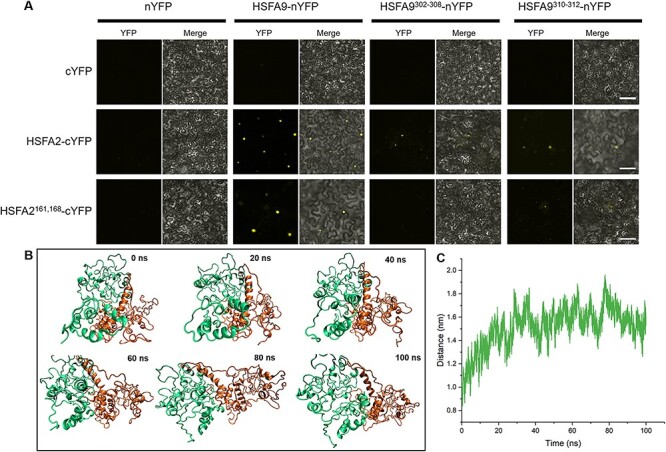
Mutating these interface residues led to decreased interaction and separation between HSFA9 and HSFA2 in the complex. (A) Bimolecular fluorescence complementation (BiFC) assay in tobacco (*Nicotiana benthamiana*) leaf cells testing protein–protein interaction between HSFA9 and HSFA2. HSFA9 HSFA2 and the mutations for the interface residues were introduced into pSYNE or pSYCE vectors, respectively, fused with *N*-terminal or *C*-terminal YFP. Reconstitution of yellow fluorescence shows positive interaction in the nucleus and absence of fluorescence in the negative control (upper panel). The middle panel shows an interaction between HSFA2 and HSFA9 and a reduced interaction between HSFA2 and HSFA9 mutation for the interface residues. Importantly, the introduction of mutations in both HSFA9 and HSFA2 residues resulted in a rare occurrence of interaction (lower panel). Bars = 20 μm. (B) Molecular dynamics simulation of the HSFA9 and HSFA2 dimer complex with mutations introduced in the interface residues. The trajectory analysis reveals that mutations in the interface residues of both HSFA9 and HSFA2 result in the dynamic separation of the dimer complex from the initial interface during the course of 100 ns simulation. (C) The displacement plot further quantifies the divergence of HSFA9 and HSFA2 due to the introduced mutations.

In all MD trajectories, no dissociation events were observed at the simulation timescale. The monomer arrangement in the protein–protein interaction complexes remained stable throughout the entire MD simulation trajectories (**Fig. 8D**). **Fig. 8E** presents a stereo view of the protein interface and interacting residues involved in the formation of a heterodimer, as determined through molecular simulation analysis. During molecular dynamics simulations, it was noteworthy that the intrinsically disordered secondary structure of both HSFA9 and HSFA2 were found to be more folded compared to regions of Alphafold2-predicted models (**Fig. 8E**). The interactions between HSFA9 and HSFA2 at the residue level were evaluated using a contact map analysis (**Figs. 8F**, **9G**). The contact map analysis showed the physiochemical characteristics of the interacting residues, revealing that 302–308, 310 and 312 regions of HSFA9 and 135–137, 161–162 and 168–169 regions of HSFA2 are the main interacting residues that contribute to their interactions. Therefore, molecular dynamics simulation is equally important to optimize and validate the accuracy of the model predicted by Alphafold2.

We further performed bimolecular florescence (BiFC) analysis to determine whether the interface in the *C*-terminal between HSFA9 and HSFA2 is responsible for their interaction. The BiFC assay revealed a positive interaction between HSFA9 and HSFA2, as evidenced by the detection of YFP signal in both HSFA9-nYFP + HSFA2-cYFP and HSFA9-nYFP +HSFA2^161,168^-nYFP pairs (**Fig. 9A**). However, when specific mutations were introduced in the *C*-terminal region (amino acid residues 302–308 and 310–312) of HSFA9, a dramatic reduction in the interaction with HSFA2 was observed (**Fig. 9A**). Furthermore, confocal microscopy confirmed that the YFP signal indicative of the interaction was localized within the nucleus, providing a molecular mechanism for the transcriptional activity of HSFA9. Comparison of sequences indicates significant variations in the *C*-terminal segments of HSFs among the well-studied HSFA9 proteins from *Helianthus annuus, M. truncatula* and *Solanum lycopersicum*. This observation implies a distinct divergence within this region among different members of the heat-shock factor family (**Supplementary Fig. S3**).

### Impact of interface residue mutations on HSFA9 and HSFA2 dimer complex dynamics

Molecular dynamics simulations were conducted to assess the consequences of mutations in interface residues on the stability of the HSFA9 and HSFA2 dimer complex. The 100-nm simulation trajectory revealed a dynamic separation of the dimer complex from the original interface due to the introduced mutations in both HSFA9 and HSFA2 (**Supplementary Video S1**). The trajectory analysis demonstrated a clear deviation of the dimer complex from its initial interaction site, indicating a disruptive effect on the protein–protein interface. The simulation snapshots illustrated distinct conformations over time, emphasizing the evolving nature of the dimer complex (**Fig. 9B**). Quantitative displacement analysis further corroborated these observations, providing a measure of the divergence between HSFA9 and HSFA2 (**Fig. 9C**). The simulation results unequivocally indicate that mutations in the interface residues lead to a notable destabilization of the protein–protein interaction, emphasizing the critical role of these residues in maintaining the stability of the protein–protein interaction.

## Discussion

The longevity of seeds, their tolerance to desiccation and ability to germination are traits of paramount importance not only for plant adaptation to stress conditions but also for agriculture and the conservation of genetic resources. Thus, identification of the underlying regulatory factors will provide valuable insights into how deterioration tolerance and seed viability are generated and maintained during seed maturation and post-harvest storage. In this study, we demonstrated that an increase in seed longevity is linked to a change in the transcriptome profile that results in the up-regulation of genes involved in dehydration, heat and hypoxia response. In addition, we also demonstrated that a functional interaction between HSFA9 and HSFA2 can improve seed longevity and seedling heat resistance.

The HSFA genes play an important regulatory role in regulating plant response to various abiotic stresses by controlling the expression of stress-responsive genes including heat shock, oxidative stress, drought and pathogen-induced proteins (Jacob et al. 2017, Ohama et al. 2017). HSFA9 is a member of the *Arabidopsis* class A HSFs which is specifically expressed in seeds. Several lines of genetic evidence indicate that HSFA9 may be involved in the complex regulatory network that controls seed longevity, either through interaction with or downstream of the other regulators (Dekkers et al. 2016, Alizadeh et al. 2021). For example, overexpression of HSFA9, which is regulated by ABI3, in sunflower (*H. annuus*) seeds leads to an increase in resistance to CDT, and tobacco plants with a loss-of-function of HSFA9 have reduced resistance to CDT (Tejedor-Cano et al. 2010). Overexpressing the sunflower HSFA9 together with the drought-responsive TF HaDREB2 resulted in positive effects on seed longevity beyond those observed with the overexpression of HaHSFA9 alone (Almoguera et al. 2009). In sunflower and tobacco, HSFA9 was found to be essential for embryogenesis and photomorphogenesis, further expanding our understanding of the diverse functions of HSFs (Kotak et al. 2007, Leprince et al. 2017, Carranco et al. 2022, Charng and Chen 2023). We show similar roles of HSFA9 in the acquisition of the thermotolerance tolerance and seed longevity in *Arabidopsis*.

The early response events induced by CDT treatment in seeds play a critical role in seedling establishment. However, most previous studies based on a smaller number of genes may hinder better insight into the underlying mechanism of seed longevity. In this study, through comprehensive comparative RNA-seq analysis, we revealed 5,042 DEGs between control and *hsfA9* groups under CDT, indicating the dynamic and complex gene regulation network. A large number of biological processes associated with controlled deterioration are involved in dehydration, heat and hypoxia, as demonstrated through GO-enrichment analysis. However, these stress-response pathways were both induced by CDT in WT and *hsfA9* mutant, making it hard to identify the key genes involved in seed longevity. Therefore, our comparative transcriptomic analysis from WT-CDT and *hsfA9*-CDT groups allowed us to identify CDT-specific differentially down-regulated genes (211) whose altered expression contributes to the reduced seed longevity in *hsfA9* lines. We found that these CDT-repressed DEGs in *hsfA9* seedlings were significantly enriched for a range of GO-terms associated with hypoxia response, oxidative stress, chromosome organization and DNA repair. Seed deterioration during the dry storage is often associated with the oxidation of macromolecules; therefore, seeds require a range of protective genes to limit oxidative stress and excessive oxidation. Chromosomal aberrations DNA strand break repair have been reported to be particularly important for seed germination and longevity (Cheah and Osborne 1978, Waterworth et al. 2015). Therefore, we deduced that these genes may potentially be involved in the regulation of seed longevity through HSFA9. It is worth noting that another heat-shock TF, HSFA2, is significantly suppressed in *hsfA9* mutants during CDT, suggesting a potential function of HSFA2 during the course of CDT.

TFs bind to specific *cis*-elements in the promoter regions of downstream genes, enabling the transcriptional regulation of these genes and the regulation of various biological processes. In connection with seed longevity, several important promoter motifs such as BPC5, ABF, REM19 and NAC were identified in CDT-induced and -repressed DEGs, indicating that these identified TF regulatory modules may function as an activator or suppressor for the transcriptional regulation during seed deterioration tolerance. Notably, previous studies identified motifs included ABF and NAC (Yoshida et al. 2015, Bizouerne et al. 2021) which share the same sequence with ABA-mediated dehydration in our study. On the other hand, promoters of 211 CDT-suppressed genes in HSFA9 knock-out lines were enriched in ABI5 and MYB related motifs, suggesting their positive roles in *Arabidopsis* seed longevity. The ABI5 and MYB motif are prominent in the promoters of the LEA and drought-resistance genes, which is essential to the establishment of seed mutation, longevity and germination (Verdier et al. 2013, Zinsmeister et al. 2016). Thus, we conclude that HSFA9 plays an important role in regulating downstream genes with ABI5 and MYB motif involved in *Arabidopsis* seed longevity.

Among the suppressed genes during CDT in *hsfA9* lines, it is worth noting that *HSFA2* plays a critical role in the induction of chaperone production and improvement of heat-stress resistance (Ohama et al. 2017). Consequently, we speculated that HSFA2 may play a regulatory role in *Arabidopsis* seed longevity and heat-response regulation. In *Arabidopsis* seeds, where the endogenous *HSFA2* and *HSFA9* were both knocked out, the seeds had a significantly reduced germination rate compared to WT and *hsfA9* seeds, indicating that HSFA2 is also involved in seed longevity. Genetic and phenotypic analyses of single knockout and *hsfA2/hsfA9* double mutants have determined that they play a crucial role in regulation of thermal resistance in vegetative tissues during heat-shock stress. Notably, in the *hsfa9* mutant, a reduction in expression levels was observed for *HSFA2, HSP17.6, HB1, NLM1, ERF019, PYL4* and *CIB1*, with *HSP101* displaying a distinct expression pattern, suggesting a unique regulatory role. This dual regulatory capability likely contributes to the intricate and context-dependent nature of HSFA9-mediated gene expression, shedding light on the multifaceted roles of these TFs in stress responses.

Based on phylogenetic relationship and structure properties, HSFs are divided into three classes, i.e. A, B and C (Fragkostefanakis et al. 2015). All the HSFs share a modular structure with an *N*-terminal DNA-binding domain for heat-shock response element of target genes, following hydrophobic heptad repeat required for oligomerizaiton and a C-terminal activation domain (AHA motif), which is specific to class A HSFs (Kotak et al. 2004, Scharf et al. 2012). Remarkably, the sequence comparison revealed significant variations in the *C*-terminal segments of HSFA9 proteins among well-studied species, suggesting a potential divergence in function within this region across different members of the heat-shock factor family (Nover et al. 2001, Verdier et al. 2013, Zinsmeister et al. 2020). To investigate whether HSFA9 is able to interact with HSFA2, we conducted a yeast two-hybrid (Y2H) assay. The results presented here demonstrate that HSFA9 and HSFA2 physically interaction with each other. It is worth noting that in a Y2H system, the bait and prey proteins are highly overexpressed and forced into the nucleus to facilitate interaction, which might potentially lead to false-positive results (Cao et al. 2009, Sjohamn and Hedfalk 2014). As a result, it is necessary to verify Y2H data using additional approaches.

The BiFC analysis provided valuable insights into the interaction dynamics between HSFA9 and HSFA2, particularly within their native state. The positive interaction observed in the BiFC assay, as evidenced by the YFP signal in various configurations, highlighted the capability of the interaction between HSFA9 and HSFA2. Intriguingly, the introduction of specific mutations in the *C*-terminal region led to a substantial reduction in the HSFA9–HSFA2 interaction, emphasizing the critical role of these residues in maintaining the protein–protein interaction. Furthermore, the molecular dynamics simulations conducted to evaluate the impact of mutations in interface residues on the HSFA9 and HSFA2 dimer complex provided valuable insights into its stability. The simulation trajectory analysis revealed a dynamic separation of the dimer complex from its initial interface due to the introduced mutations in the C-terminal of HSFA9 and HSFA2. These combined results contribute to a comprehensive understanding of the molecular interactions and sequence variations governing the functionality of HSFA9 in response to cellular stress.

Protein structures can provide invaluable information for both protein biological function and protein–protein interactions. However, using conventional techniques such as X-ray diffraction and cryo-electron microscopy to characterize the structural features of protein–protein interaction can be costly, time-consuming and technically challenging. Recently, the deep learning-based AlphaFold2 shows remarkable success in accurately predicting the 3D structures of proteins based on their amino acid sequences (Jumper et al. 2021). Consequently, molecular docking experiments based on the model of HSFA9 and HSFA2 were performed to investigate their interaction. The complexes generated from docking analysis were selected and subsequently validated through 100 ns of MD simulations for minimizing and optimizing their geometry. MD simulation is a powerful tool for analyzing the structure and interaction dynamics of proteins with their molecular partners and surrounding environment (Wang et al. 2019). This technique allowed us to examine the conformational dynamics and functional characteristics of protein at an atomic level of detail (Tang et al. 2021).

Interestingly, although Alphafold2 returns full protein predictions, we noticed that some fragments with intrinsically disordered regions (IDRs) had very low confidence. On the contrary, the disordered region of the predicted models from Alphafold2 were further improved and refined by applying molecular dynamics simulation. Here, we show that the combination of machine-learning-based models from AlphaFold2 and state-of-art physics-based refinement by applying MD further improves the accuracy of protein structure.

## Conclusion

**Fig. 10:**
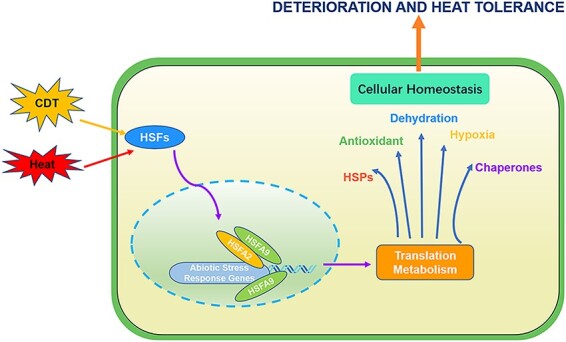
Integrated regulatory model of HSFs in deterioration and heat stress response. HSFA9 leads to the activation of downstream targets, including HSPs, antioxidant enzyme genes, dehydration-responsive genes and genes involved in hypoxia response and chaperones. HSFA9 takes a central role in this regulatory network, interacting with HSFA2 and binding to the promoters of their downstream regulatory genes during heat shock stress. This integrated regulation enhances the plant’s capacity to develop tolerance to both deterioration and heat stress.

Taken together, we showed that CDT has an inhibitory effect on *Arabidopsis* seed vigor and germination and analyzed the genome-wide transcript expression changes based on RNA-seq data. By performing comparative transcriptome analysis, we identified 1,731 CDT-induced and 211 CDT-suppressed DEGs that were closely related to the regulation of seed vigor and germination. Furthermore, function enrichment analysis of the DEGs regulated by CDT revealed that genes involved in hypoxia, oxidative stress and DNA repair were the most likely candidates contributing to CDT-mediated seed longevity and germination vigor. Further, significant enrichment of *cis*-element ABI5 and MYB in the promoter regions of these CDT-suppressed genes suggests the involvement of these genes in *Arabidopsis* seed longevity. Moreover, with the combination of molecular dynamic simulation studies, molecular docking increases our knowledge of the interaction between HSFA9 and HSFA2, and expands our understanding of the unique properties of HSFA9 structure in connection with its functions. In summary, HSFA9 plays a pivotal role in orchestrating the activation of key downstream targets critical for stress response, encompassing HSPs, antioxidant enzyme genes, dehydration-responsive genes, chaperones and genes involved in hypoxia response (**Fig. 10**). This integrated regulatory mechanism significantly contributes to enhancing the plant’s ability to cultivate tolerance against both deterioration and heat stress, highlighting the multifaceted and coordinated nature of HSFA9-mediated stress responses.

## Materials and Methods

### Plant materials and growth assay

The *Arabidopsis thaliana* Colombia-0 (Col-0) ecotype was used in this study.

T-DNA insertion lines SALK_063950 (*hsfA9*) and SALK_008978 (*hsfA2*) mutants were obtained from the Arabidopsis Biological Resources Center (ABRC, Columbus, OH, USA). The *hsfA9/hsfA2* double mutants were generated from a cross between *hsfA9* and *hsfA2*. To complement the *hsfA9* mutant, a full-length HSFA9 (AT5G54070) cDNA was amplified and ligated to binary vector pBI121 under *HSFA9* promoter. The 2.0kb *HSFA9* promoter was amplified from genomic DNA before the start codon. For overexpression constructions, the full-length cDNA of HSFA9 and HSFA2 were cloned into the pCAMBIA1300 binary expression vector. The resulting vectors were transformed into Agrobacterium *tumefaciens* strain GV3101. Transformation of *Arabidopsis* plants was performed using the floral dipping method (Clough and Bent 1998). The homozygous identity of T-DNA insertion mutants and transgenic lines was confirmed by PCR assay in the T2 and F2 plants, respectively.

*Arabidopsis* seeds were surface-sterilized using the method described previously (Wang et al. 2015). The seeds were planted in a medium containing 1% agar, 0.5× Murashige and Skoog salts, 1% sucrose and a pH of 5.8. After incubating the plates at 4°C for 2 days, they were transferred to a growth chamber kept at 23°C under a 16-h light/8-h dark cycle.

The germination rates were calculated every 2 h based on three separate biological replicates. Approximately, 30 young seedlings from the normal and CDT conditions were collected from each of the three biological replicates after 6 h germination. The young seedlings were rapidly frozen by liquid nitrogen and stored at −80°C before extracting RNA.

### CDT of seeds

The CDT was conducted according to the previous method (Prieto-Dapena et al. 2017) with slight modification, as well as for measuring basal thermotolerance. Dry *Arabidopsis* seeds were rehydrated for 1 h before exposing to high humidity and temperature condition. To perform CDT, moisture content was increased to 36% by imbibition in saturated calcium chloride solution for 3 days. The seeds were then dried and placed in sealed bags before being incubated at 45°C in a water bath. After these treatments, the seeds were used to analyse vigor, germination percentage and RNA-seq.

### Thermotolerance assays of seeds and seedlings

The basal thermotolerance of seeds were conducted by incubation of the Eppendorf tubes containing the seeds in water baths at 55°C for 20, 40 and 60 min. The treated seeds were further used to assess the germination rate. For the heat-shock tolerance assay, seedlings were treated at 37°C for 1 h and then allowed to recover at 22°C for 2 days.

A subsequent stress treatment at 42°C for 45 min was performed immediately thereafter. After heat treatments, plants in Petri dishes were then cultured under normal growth conditions at 22°C and were photographed for 5 days. The Petri dishes were sealed with transparent film during heat-shock treatment.

### RNA sequencing and analysis

Total RNA was isolated and purified using Trizol reagent (Invitrogen, CA, USA) following the manufacturer’s procedure. cDNA libraries were prepared from mRNA using the TruSeqTM Stranded mRNA sample preparation kit (Illumina, San Diego, CA, USA) then sequenced on an Illumina HiSeq 4000 (Illumina Inc.) with 150-bp paired-end reads. Raw reads were filtered using the fastp software with default parameters (Chen et al. 2018). The *Arabidopsis* genome (TAIR version 10) was used as a reference for aligning the filtered sequencing reads, and Bowtie2 software (Langmead and Salzberg 2012) was utilized for all subsequent analyses. The gene expression levels were determined using the fragments per kilobase of the transcript per million mapped reads (FPKM) method. Heat maps were drawn using the Pheatmap package (https://cran.r-project.org/web/packages/pheatmap/) in R and were clustered using Pearson correlation distance. For DEG analysis, DEseq2 package was used to identify DEGs between two groups (Love et al. 2014). DEGs were identified as genes with an adjusted P-value less than 0.01 and a fold change greater than 3. GO-enrichment analysis was conducted using the R cluster Profiler package (Yu et al. 2012). A *k*-means approach (R package ‘Mfuzz’) was used to cluster genes with similar dynamics of expression during CDT (Kumar and Futschik 2007). Multiple values of *k* were tested, and *k* = 6 was selected as the optimal number of clusters based on the highest gradual membership values.

### Co-expression network construction and visualization

Co-expression networks were established through the application of the WGCNA package (v1.29) in R (Langfelder and Horvath 2008). The collective gene expression values from both control and CDT conditions were employed to identify the comprehensive set of gene expression differences. From these DEGs, those exhibiting non-zero variations and a missed rate of less than 10% were selected and transformed into an adjacency matrix, representing connection strengths. Following the software instructions, calculations and visualizations were performed for the module eigengene expression, adjacency matrix heatmap, module–trait relationships and other pertinent parameters and results.

### Quantitative real-time PCR validation of RNA-seq data

Validation of the significant DEGs and HSFA9 target genes was conducted through RT-qPCR. Actin2 served as the internal control, and the primer details are provided in **Supplementary Table S1**. RT-qPCR analysis for each gene was carried out in three biological replicates, each with three technical replicates. The 2 − ΔΔCT method was employed to calculate the relative gene expression levels.

### Bimolecular florescence analysis and confocal microscopy

HSFA9, HSFA2, HSFA9^302−308^, HSFA9^310−312^ and HSFA2^161,168^ were cloned into the pSYNE and pSYCE vectors with a YFP conserved domain to generate *N*-terminal or C-terminal fusion proteins, respectively. Each plasmid was introduced into *A. tumefaciens* GV3101 and infiltrated into 4-week-old *Nicotiana benthamiana* leaves as described previously (Ham et al. 2023).

The infected leaves were imaged after 48 h using a Leica SP8 confocal laser scanning microscope (Leica Microsystems, Buffalo Grove, IL, USA).

### Tetrazolium staining

Seeds were stained with 1% solution of 2,3,5-triphenyltetrazolium chloride (Sigma) and incubated for 8 h in root temperature. The tetrazolium salts were converted into highly colored end products known as formazans through a process of metabolic reduction. Photographs of the stained seeds were taken using a Zeiss Axioplan upright microscope.

### Promoter *cis*-element analysis

The promoter sequences (2,000 bp sequences upstream of the start codon) of 1,731 and 211 genes were collected from the *Arabidopsis* genome (TAIR version 10) and submitted to the discriminative regular expression motif elicitation (DREME) (Bailey 2011). The most overrepresented motifs in these genes were identified using default parameters. The identified DNA Motifs in *Arabidopsis* were then subjected to a homology search using the motif comparison tool Tomtom (Gupta et al. 2007) to identify the best matching motifs.

### Protein–protein docking

The 3D structures of HSFA9 and HSFA2 were predicted through AlphaFold2 (https://www.alphafold.ebi.ac.uk/, accessed on 10 September 2022) (Jumper et al. 2021). To study the interaction between HSFA9 and HSFA2, molecular docking between the two proteins was performed through the ZDOCK v3.0.2 (Pierce et al. 2014). The top 10 docking models were visually inspected and selected for further molecular dynamic simulation.

### Atomistic molecular dynamics simulation

Initial structural models of the HSFA9 and HSFA2 complex were built with ZDOCK mentioned above. GROMACS 2019.3 was used for all atomistic molecular dynamics simulations, with the AMBER99SB, all atom force field and the TIP3P water model (Pronk et al. 2013). The simulations were conducted at 298 K using the Berendsen thermostat with a time constant of 0.1 ps. The pressure was maintained at a constant value of 1 bar through the use of a semi-isotropic coupling constant of 1.0 ps and a compressibility = 4.5 × 10^−5^ bar^−1^. The integration time step was set to 2 fs. All the bond length was constrained using the LINCS algorithm (Hess et al. 1997). A distance of 1.2 nm was used as the cutoff for van der Waals and electrostatic interactions. Periodic boundary conditions and the particle-mesh Ewald (PME) algorithm (Essmann et al. 1995) were employed to handle long-range electrostatic effects. Coordinates were saved every 2 ps for the consequent analysis. All MD simulations were conducted for 100 ns over five independent runs. The MD trajectories were visualized, and all snapshots were created using VMD software version 1.93 (Humphrey et al. 1996).

### Yeast two-hybrid screen

The Y2H experiments were carried out using Matchmaker library construction and screening kits following the manufacturer’s instructions (Clontech, Palo Alto, CA). First, the cDNA for *HSFA9* and *HSFA2* was inserted in frame into the GAL4 DNA-binding domain in vector pGBKT7 (BD) and the GAL4 activation domain in vector pGADT7 (AD), respectively. The resulting plasmids were co-transformed into *Saccharomyces cerevisiae* AH109 cells. Transformed yeasts containing both BD and AD vectors were selected on synthetic-defined (SD) medium lacking Trp and Leu. Protein interactions were assayed by growing the transformed yeasts on SC medium lacking adenine, His, Trp and Leu.

## Supplementary Material

pcad164_Supp

## Data Availability

The RNA-seq project has been deposited in the National Center for Biotechnology Information (NCBI) under the BioProject accession no. PRJNA930172. Raw sequencing data have been deposited in Sequence Read Archive (SRA) database as SAMN32989484 to SAMN32989495. All relevant data are included within the manuscript.
