# Supplementary material for: Arabidopsis HSFA9 Acts as a Regulator of Heat Response Gene Expression and the Acquisition of Thermotolerance and Seed Longevity
Source: Plant Cell Physiol. 2023 Dec 20;65(3):372–89. doi: 10.1093/pcp/pcad164 (PMC11020252; doi:10.1093/pcp/pcad164)
Supplement: pcad164_Supp [file pcad164_supp.zip › suppl_data/pcp-2023-e-00280-File014.pdf]

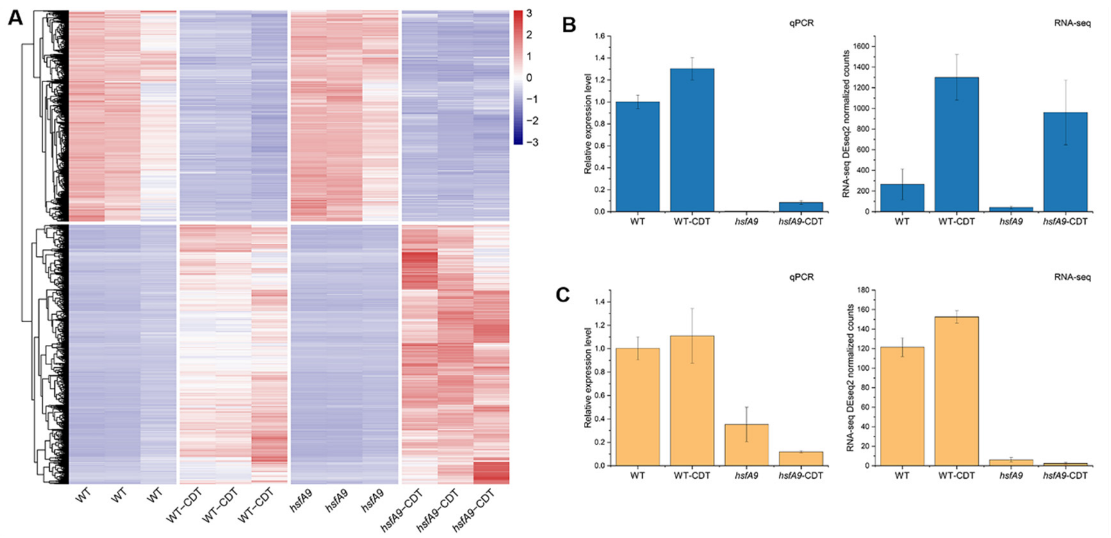

Supplementary Fig. S1. Gene expression profiles of seed in response to controlled deterioration.

(A) Heatmap showing expression profiles of differentially expressed genes (DEGs) in the four data sets. The color scale represents expression level, firebrick indicates high expression, and navy blue indicates low expression.

(B) RT-qPCR of HSP17.6 (left panels) and the corresponding RNA-seq DEseq2 normalized counts (right panels).

(C) RT-qPCR of PAR1 (left panels) and the corresponding RNA-seq DEseq2 normalized counts (right panels).

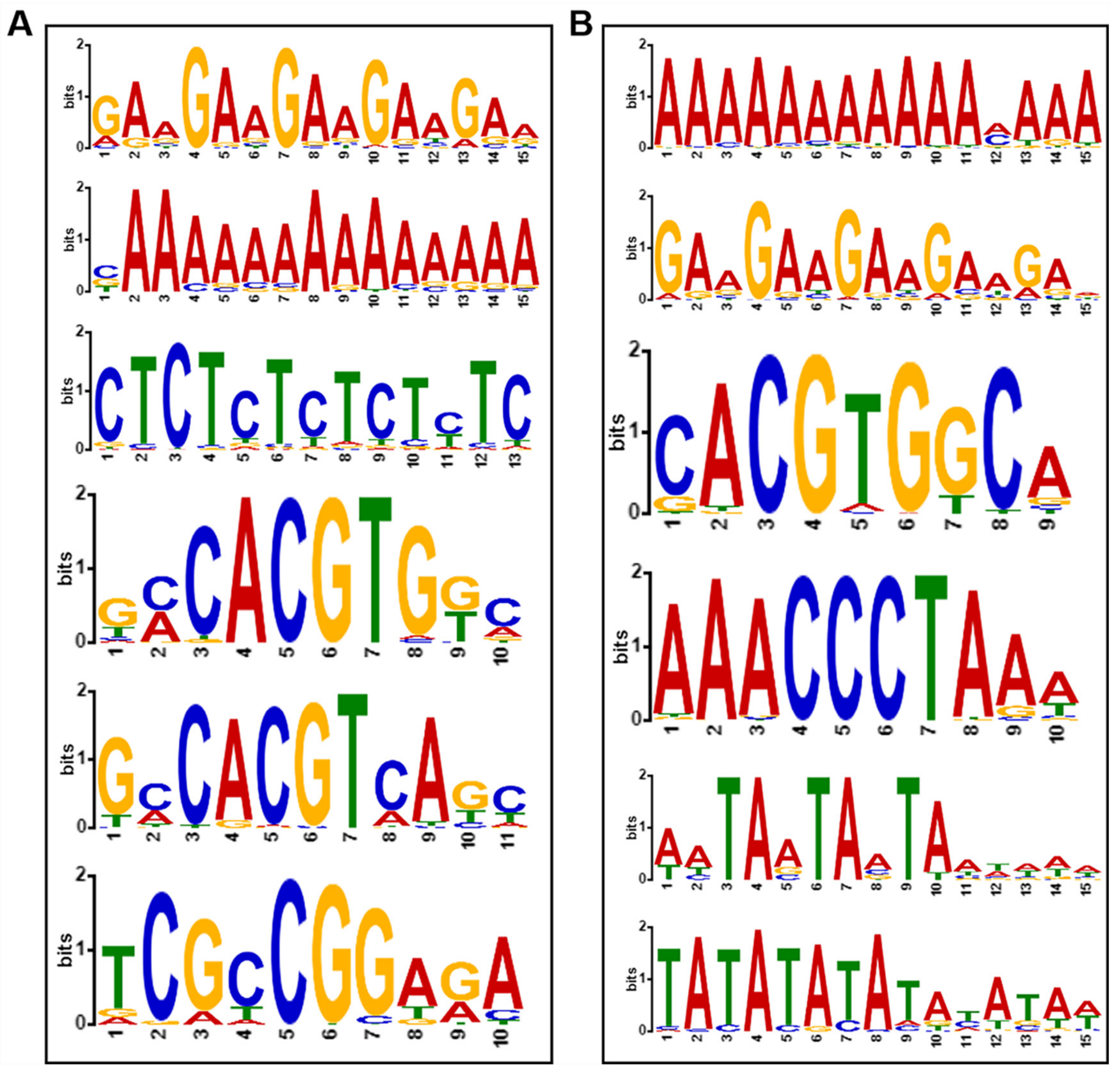

Supplementary Fig. S2. The cis-elements enriched in the promotors of CDT-induced (A) and -suppressed genes (B). The 2.0 k bp promotor sequence of these genes were collected from the Arabidopsis genome (TAIR10) and analyzed by DREME and Tomtom to identify the overrepresented motifs.

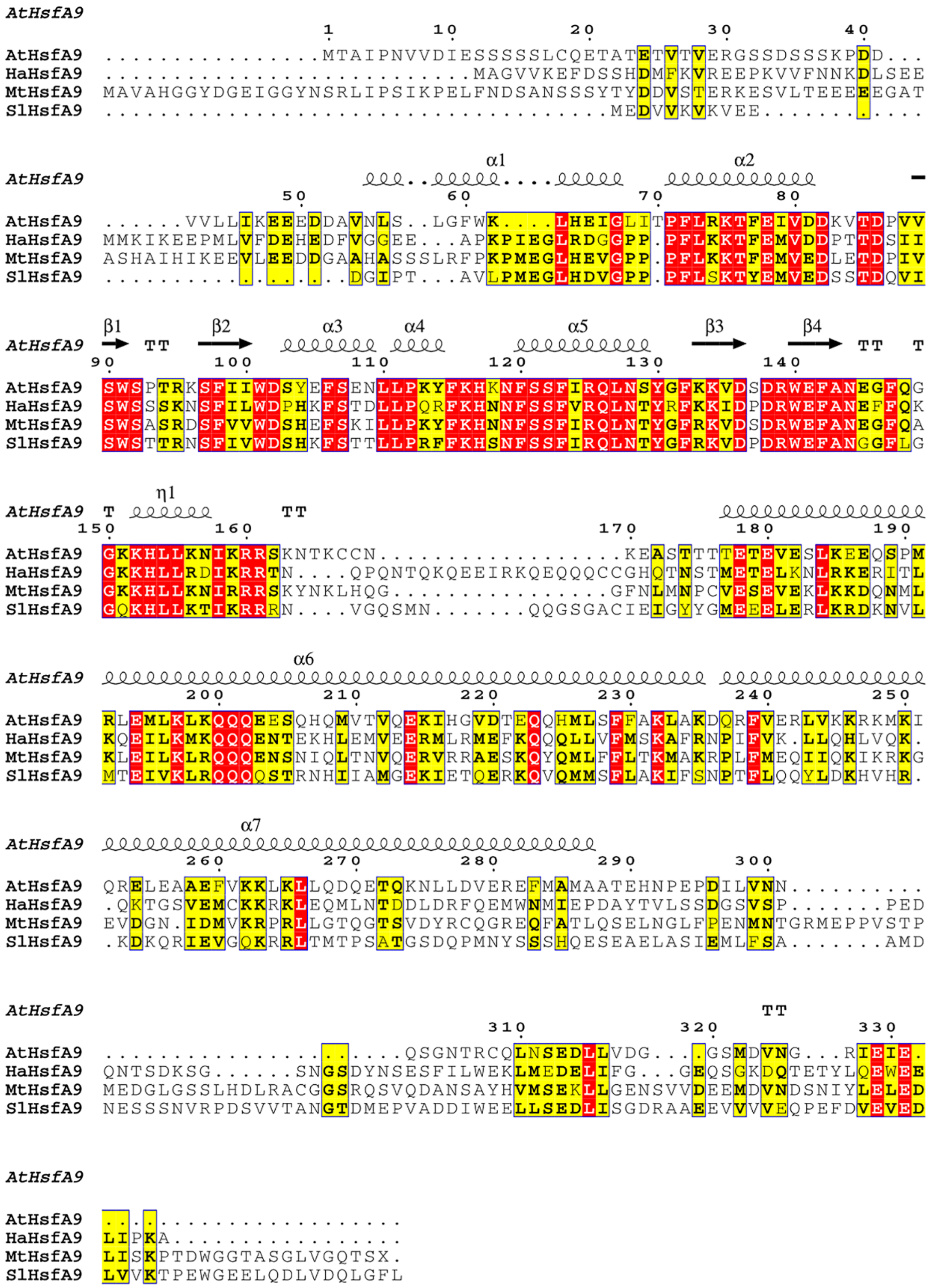

Supplementary Fig. S3. Multiple sequence alignment of HSFA9 performed using ClustalW. Comparison of sequences indicates significant variations in the C-terminal among HSFA9 proteins from *Arabidopsis thaliana*, *Helianthus annuus*, *Medicago truncatula* and *Solanum lycopersicum*.

Supplementary Table S1. Primers used for RT-qPCR detection of HSFA9 target genes and validation of RNA-seq

Gene name	Forward 5'-3'	Reverse 5'-3'
ACTIN2	TGCACCAGAAGTCTTGTTCC	TTATTGCTTGGTGCAAGTGCTG
HSFA2	AAGAAACGGTGACGTTTACTGG	AATCCCACACCACAAAGCTG
HSP17.6	CGTAACAACCCTTCAAGAGC	TCATAAACTTCCCCATCCTCC
PAR1	TGTCAGAGACTAACGCAAGC	TCAACCTCCGAACTTCATGTC
HB1	CCAATTCCTGCTGAGCAAAATC	GTCTCCAACAATGCATACTTGG
ERF019	GTCAGTCAAAGTACAAAGGAATCC	CTGGATAGATCTCGGAGAAGT
NLM1	TTGATCGCGGAGTTTCTTGG	AAACTCCATCGTAAACGCCTG
PYL4	ATTCAAGAGATCTCCGCTCC	GTCAACGAAGTCACAAGTCTC
CIB1	ATGAATGGAGCTATAGGAGGTG	TCTGCTATGCTGTGACTATCAG
HSP101	ACTTCAGACCAGAGCTCTTG	ATAGGCCTAGCACCATACAC

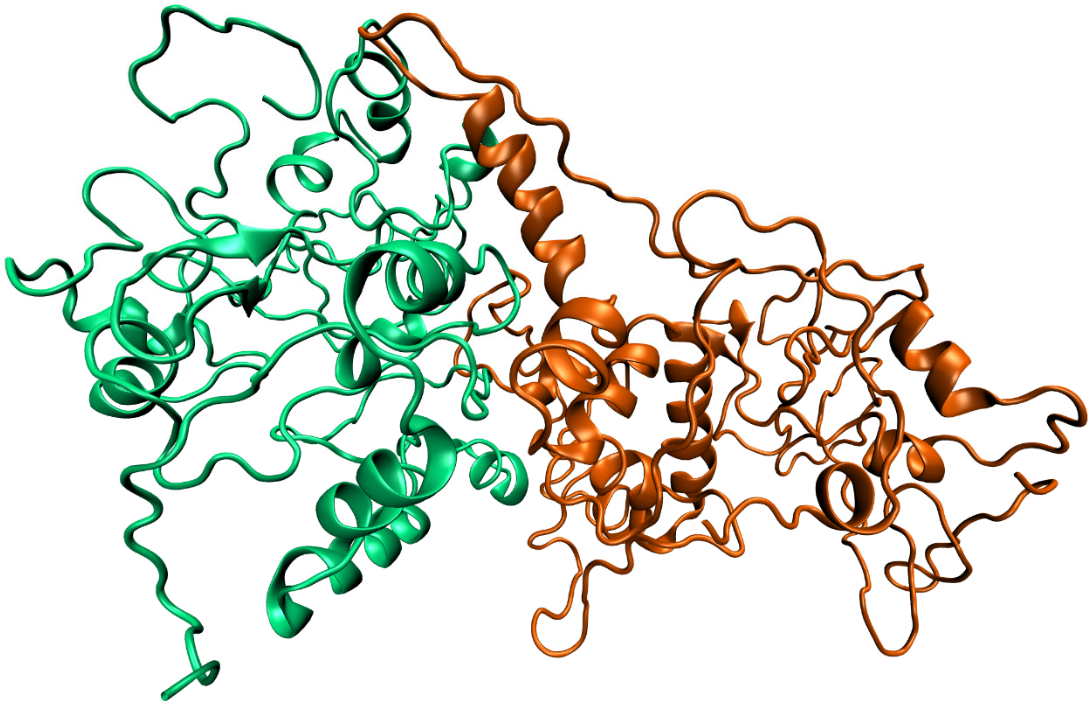

Supplementary Video S1. Dynamics of HSFA9 and HSFA 2 dimer complex in 100 nm simulation. A notable dissociation of the dimer complex from its initial interface, resulting from the introduced mutations in HSFA9.
